# Errors according to the number of registered markers used in navigation-assisted surgery of the mandible

**DOI:** 10.1186/s13005-019-0190-z

**Published:** 2019-02-09

**Authors:** Young-Eun Hwang, Sang-Hoon Kang, Hang-Keun Kim

**Affiliations:** 1 Department of Biomedical Engineering, College of Health Science, Gachon University, Incheon, South Korea; 2 Department of Oral and Maxillofacial Surgery, National Health Insurance Service Ilsan Hospital, Goyang, Republic of Korea; 3 Department of Oral and Maxillofacial Surgery, College of Dentistry, Yonsei University, Seoul, Republic of Korea; 4 Department of Health Sciences and Technology, GAIHST, Gachon University, Incheon, South Korea

**Keywords:** Navigation surgery, Image-guided surgery, Mandible, Registration

## Abstract

**Background:**

The aim of this study was to evaluate the accuracy of navigation according to the number of markers in terms of target registration errors (TREs) at each anatomical location during the registration process of the navigation system for the mandible.

**Methods:**

The TREs were measured in five different experiments, varying only in the number of registration reference markers, which ranged from three to seven. To measure the TREs according to the number of registration reference markers, two experimental navigation devices were used: *1) Cbyon navigation surgery equipment 2) Polaris optical tracker.* Both experiments were conducted to obtain the TREs at the anatomical locations of the mandible according to the number of registration markers during the navigation process. Statistical analysis was performed using the SPSS 23.0 software.

**Results:**

At all anatomical locations, errors were 2 mm or less. Further, significant differences in the target errors measured by the Cbyon system were found according to the number of registration markers. Significant differences in the target errors measured by the Polaris optical tracker were found according to the registration markers at the posterior border only. In both groups, the target errors did not decrease as the number of registration markers increased.

**Conclusions:**

This study demonstrates that an increase in the number of registration markers is not associated with a decrease in the TRE, and that a specific number of registration markers could reduce the TREs at each anatomical site. It is important to determine the minimum number of image registration markers at which the smallest TRE would be observed for different surgical sites.

## Background

The recent development of medical imaging technology focuses on obtaining real-time information, as well as data visualization [1, 2]. Research and development aimed at increasing access to real-time data to enable faster diagnosis and treatment, has become important [1, 3]. Access to real-time data is especially important in surgical environments where two-dimensional (2D) or three-dimensional (3D) reconstructed images can be accessed in real time [4]. Such access has been reinforced by the increased number of available surgical navigation systems and computer-assisted surgical systems using augmented reality [5].

During surgery using computer-assisted navigation, surgeons use preoperative imaging data in real time to view anatomical structures that are difficult to grossly examine [3, 6]. It allows for a minimally invasive surgery that accurately adheres to the treatment plan established prior to the surgery [6]. The range of application of this technology is gradually expanding in various surgical fields, including the fields of oral and maxillofacial surgery [7].

Image-guided computer-assisted surgery using the imaging data of patients obtained from a dataset collated before surgery is performed under the guidance of a navigation system connected to a computer. During surgery, the navigation system tracks the surgical tools to obtain the anatomical location of the surgical sites and surgical tools. However, even image-guided computer-assisted surgery is subject to errors, which must be minimized [8].

In image-guided computer-assisted surgery, computer-generated images such as computed tomography (CT) scans are overlaid with the actual anatomical environments of the patients. This technique is widely used in craniofacial surgeries, but to a limited extent in mandibular surgery [9]. Although anatomically connected to the cranium through the temporomandibular joint, the mandible moves independently of it. The soft tissue of the mandible has a small area and is subject to frequent movements of the lips and cheek, compared with other cranio-maxillo-facial areas.

During the navigation-assisted surgery of the oral and maxillofacial region, the cranio-maxilla including the upper and lower jaws, continuously move for the surgeon’s convenience or needs. Its position must be maintained even after osteotomy to prevent the loss of its reference point. Hence, a frameless fixator navigation is required. Additionally, the RF located on the jawbone must not interfere with the surgical site or the handling of surgical tools, while being recognized by the navigation system in real time.

In mandibular surgery using computer-assisted navigation, special sensor frames are attached to the mandible to allow the surgeon to optically track its location, thereby adjusting the independent and continuous movement of the mandible during surgery. To increase the accuracy of navigation, the location of the mandible is directly monitored rather than being monitored relative to the location of other cranial structures. During this process, the sensor frame must be affixed close to the surgical site such that the surgeon can monitor the positional changes. Once the frame is affixed, the registration process, in which the patient’s actual location coordinates with the lower jaw are matched against the coordinates on the rendered CT scan of the mandible, begins.

During the registration process, a number of markers that overlay two coordinates are selected. Several errors can arise during the image-guided computer-assisted surgery. Errors that occur during the image-guided surgery can be classified as technical, image, registration, application, and human errors. Registration errors arise during the process of coupling image data to the patient’s anatomical parts. Registration errors are typical errors in image-guided surgery. A paired fiducial point is the point at which a given image data matches the patient’s anatomical parts.

In the head and neck area, template marker-based registration using bite splints may be performed using a registration method involving noninvasive markers [10, 11]. An external registration frame that uses fiducial markers in the form of a denture-fixation acrylic template, and a template mouth piece around the jawbone, have been reported to exhibit similar registration accuracies as invasive markers that are implanted on the bone. The latter does not require an invasive procedure such as the implantation of screws used as markers. However, it has the disadvantage of requiring a marker template prior to the imaging process and having the patient bite onto the device while CT scans are being obtained.

It is important to reduce registration errors. As they are affected by the number of registration points as error factors, it is necessary to measure the errors and determine the appropriate number of registration points to reduce patient burden during image-guided surgery using a navigation system. Because target registration errors (TREs) reflect the accuracy of the navigation equipment in representing the target anatomical markers, this study aims to evaluate the accuracy of navigation according to the number of markers in terms of TREs at each anatomical location during the registration process of the navigation system for the mandible.

## Methods

### Mandibular model for measurement of TREs

**Fig. 1:**
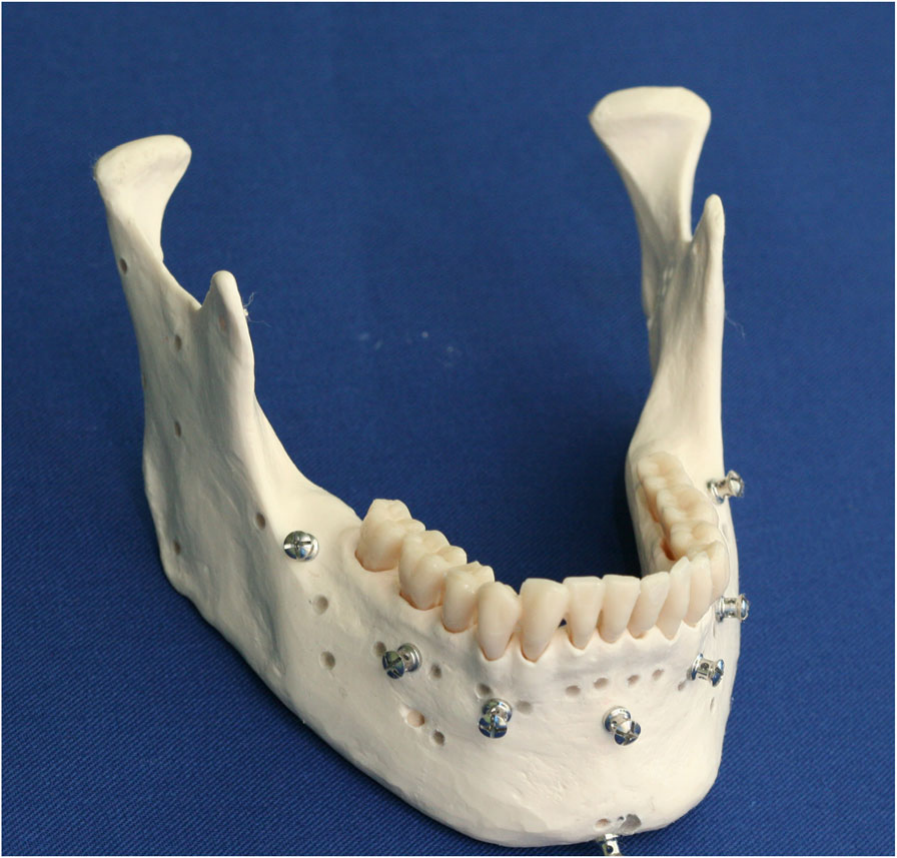
Mandibular models with markers placed. Anatomical sites are marked as holes for TRE measurements

Five mandibular (3B Scientific GmbH, Germany) A20 skull models were used in the experimental model. None of the models exhibited distinct surface defects. Screws implanted in clinically accessible locations on the models were used as the registration reference markers. In this study, 2.0-mm titanium intermaxillary fixation screws (Synthes, West Chester, PA, USA) were used as markers during the registration. An identical number of screws was implanted on all the models. A total of seven markers were implanted: one at a location 6 mm below the root apex of the mandibular central incisor, two on the alveolar bone above the mental foramen, two on the mandibular body of the mandibular first molar, and one on each ramus. To recognize the sites where errors were to be measured using different overlaying methods, small holes were created on the condylar head and neck, coronoid process, sigmoid notch, posterior border, antilingula, and angle of the mandible to measure the TREs at the anatomical markers, and filled with wax to mark the sites of error measurement (Fig. 1).

### Measurement of TREs with the two navigation devices: Medical navigation system and laboratory navigation equipment

**Fig. 2:**
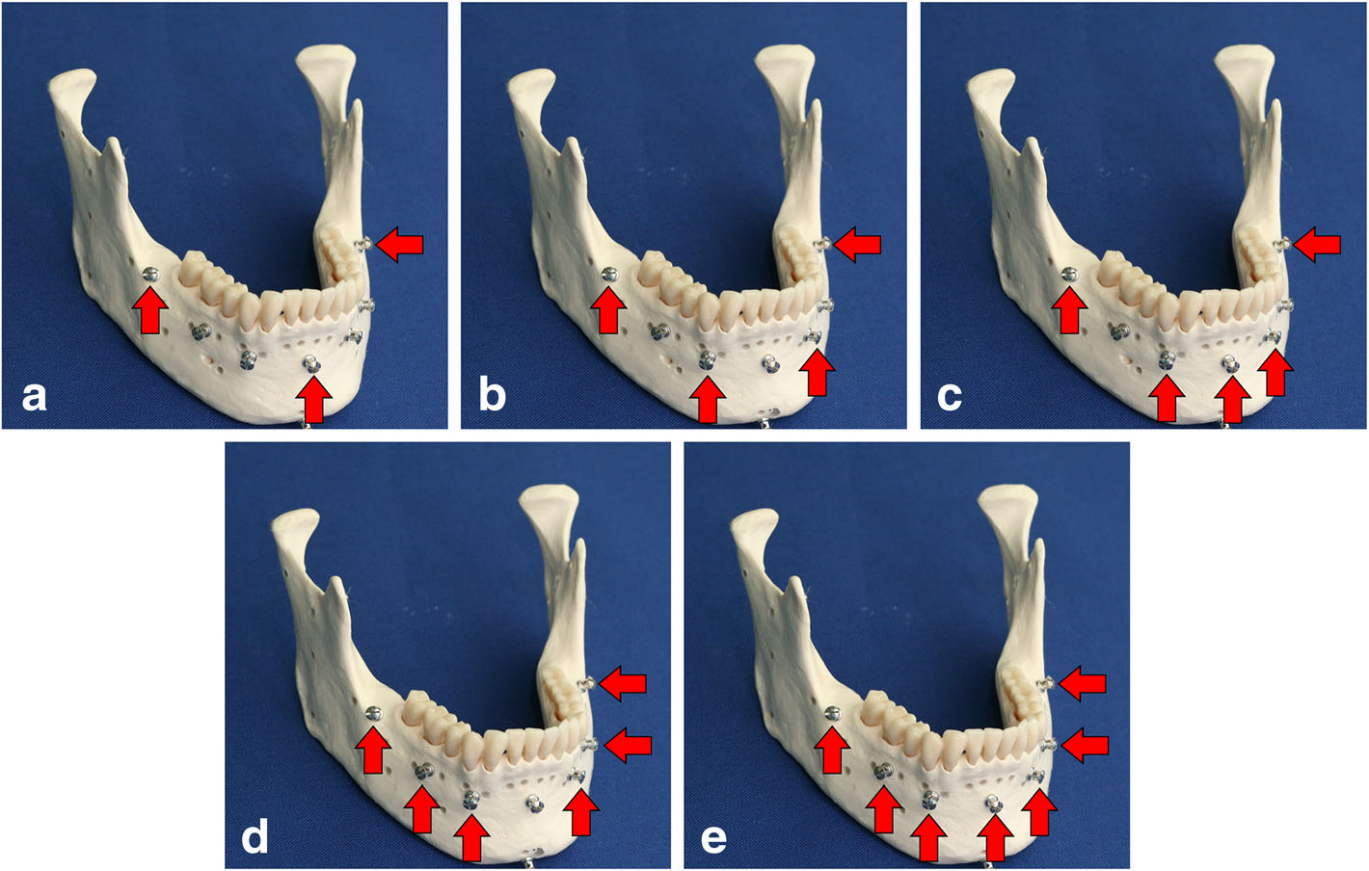
Locations of the five registration reference markers**. a** 3-point markers. **b** 4-point markers. **c** 5-point markers. **d** 6-point markers. **e** 7-point markers

In this study, TREs were measured at various anatomical locations to investigate the accuracy of navigation according to the number of registration reference markers for each model. TREs were measured in five different experiments, varying only in the number of registration reference markers ranging from three to seven. Screws were implanted on clinically usable and accessible points to mark the locations of the markers. Five methods were selected after considering the symmetry and area (Fig. 2).

Two experimental devices were used to measure the TREs according to the number of registration reference markers as described above. The Cbyon navigation surgery equipment (Cbyon, Inc., California, USA) and Polaris optical tracker (Northern Digital Inc., Waterloo, Canada) were used to measure the TREs.

### TREs according to the number of markers registered using Cbyon navigation

The CT scans of the experimental jaw models were obtained as in real surgical procedures. SOMATOM Sensation (64-slice CT, Siemens, Germany) was used to obtain the CT scans. Data were obtained from 0.7-mm-thick slices. Digital imaging and communication in medicine (DICOM) files of the obtained CT scans were imported onto the CBYON SuiteTM navigation system (Cbyon, Inc., California, USA). A reference frame was affixed to the mandibular anterior mentum of the mandibular model.

**Fig. 3:**
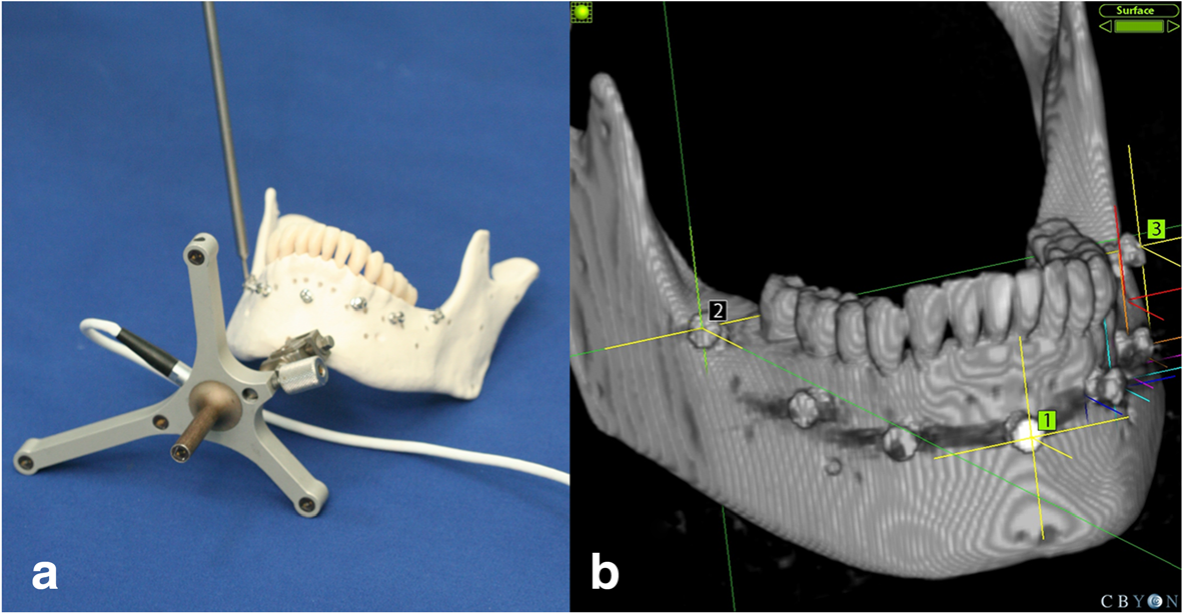
The registration process using Cbyon navigation system. **a** registration in the mandibular model. **b** registration in the Cbyon navigation system (e.g., 3-point)

On the image data imported onto the navigation system, the implanted marker screws were used to set three to seven registration reference points for the navigation registration. A probe was positioned on the marker screws to ensure that their locations matched those on the CT scans displayed on the monitor, after which registration was started. The registration process involved matching the reference points of the screw heads on virtual images against the actual markers on the mandibular model using a probe (Fig. 3).

Ultimately, the coordinates on the CT scans were matched against the actual coordinates on the mandibular model. Following the registration process, target points were marked on the condylar head and neck, coronoid process, sigmoid notch, posterior border, antilingula, and angle of the mandible to show the anatomical markers for the TRE measurement.

**Fig. 4:**
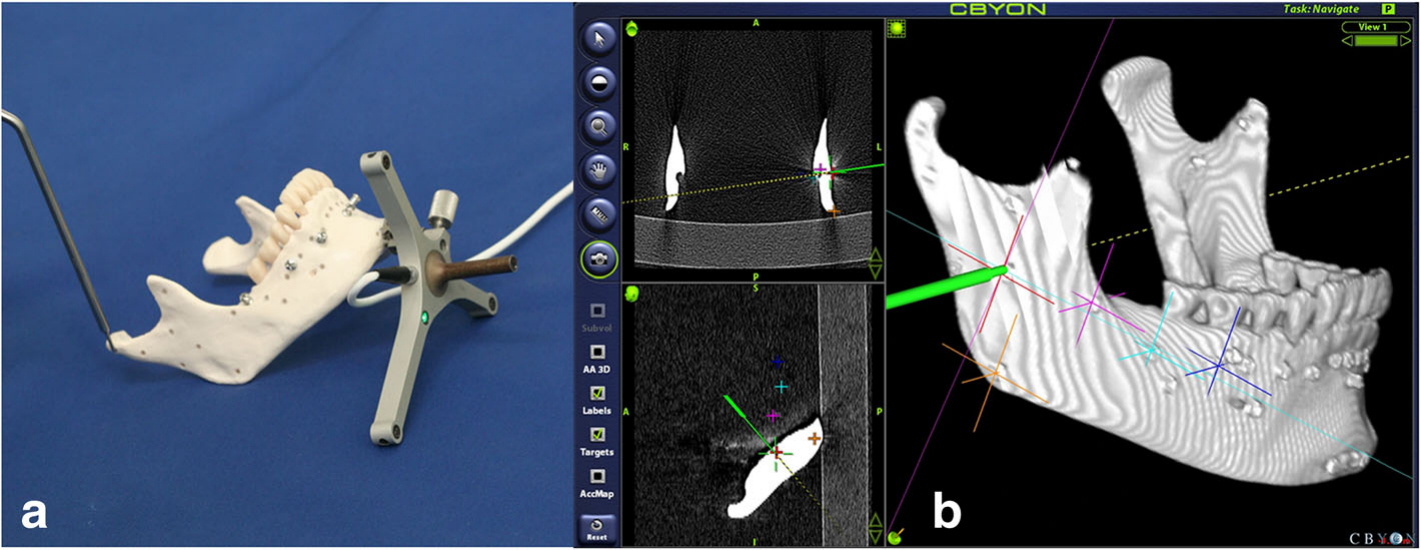
TRE measurement of mandible model in the Cbyon navigation system. **a** Probing for TRE in the mandibular model. **b** TRE measurement in the Cbyon navigation system (e.g., antilingula)

In this study, the number of references required in the registration process was changed from three to seven to evaluate the accuracy of navigation under each condition. The TREs were measured at all target points using a probe. Therefore, the TREs were measured under five different conditions varying in the number of registration reference markers for each of the five models, three times for the left and right sides, separately (Fig. 4).

Each anatomical measurement point was set as the target. The error was measured when the probe was positioned at an anatomical measurement point on the model, at a unit of 0.1 mm using Cbyon. The errors were measured 10 times per anatomical site for each of the five models. One hundred TREs were obtained for each anatomical site at varying numbers of markers.

### Polaris optical tracker and TREs measured according to the number of registration markers using the 3D slicer medical image software

**Fig. 5:**
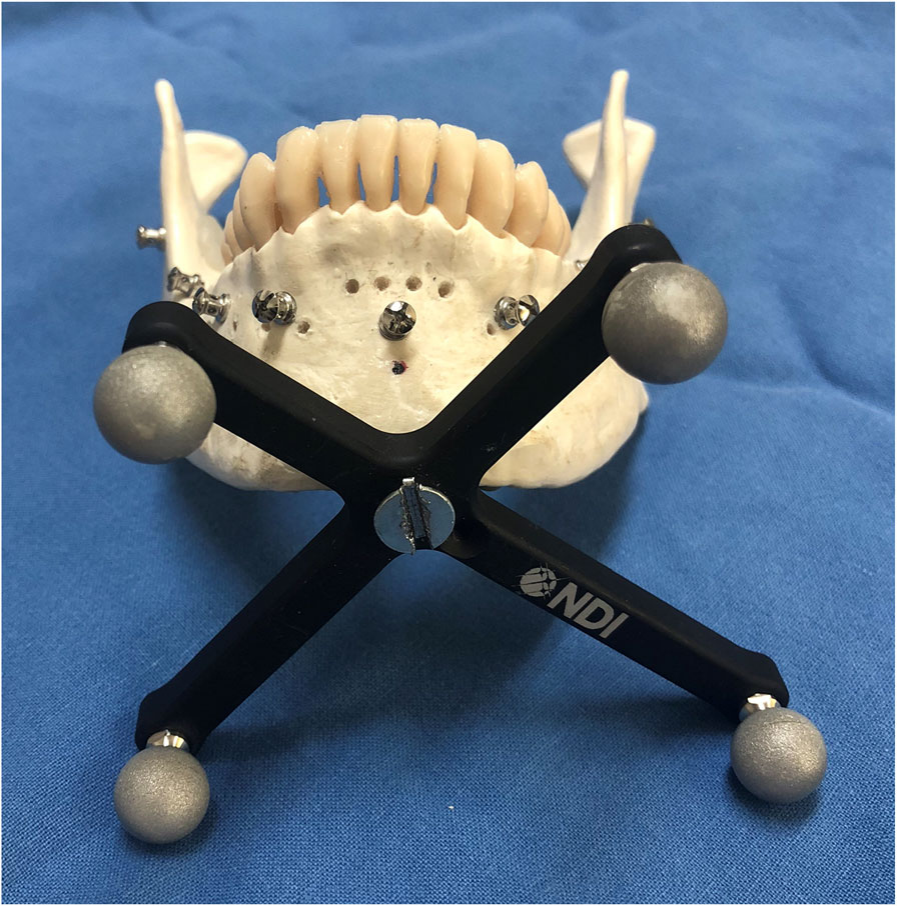
Mandibular model with an RF for the Polaris optical tracker

The optical tracking system (Polaris, Northern Digital Inc., Waterloo, Canada), and 3D Slicer (open-source medical software) were used to verify the reliability of the experiment. For a tracking system for obtaining the spatial coordinates of a mandibular model, Polaris (Northern Digital Inc., Waterloo, Canada) was used. Navigation registration based on changes in the position of the Polaris probe relative to the reference frame (RF) attached to the mentum of the mandibular model was performed. The RF continuously tracked the patient’s position and movements. It was affixed in the mandible because the mandible is prone to frequent movements. The RF was fixed in the mentum, which is the center of the mandible, to avoid tooth damage (Fig. 5). In addition, the RF and the optical sensor attached to the probe were positioned to always point toward the position sensor, and any interference in their paths due to other objects was prevented.

**Fig. 6:**
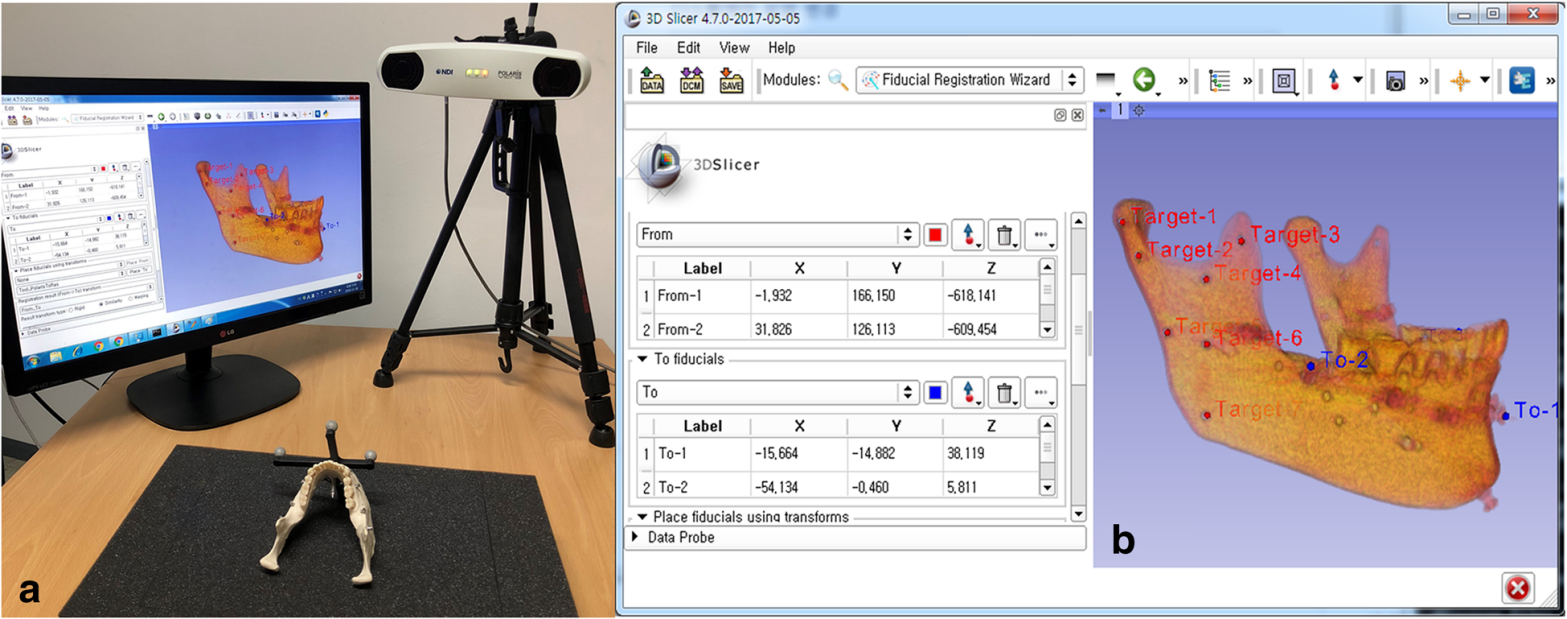
Registration within 3D Slicer with Polaris optical tracker. **a** registration in the mandibular model. **b** registration in the Polaris optical tracker and 3D Slicer software (e.g., 3-point)

The 3D Slicer is an open-source software platform for medical imaging computing that allows multipurpose visualization and provides advanced features for various applications in medical imaging. The 3D Slicer is connected to the image-guided therapy devices through OpenIGTLink. OpenIGTLink, which is a TCP/IP network communication protocol, can be used to wirelessly share various kinds of tracking systems and data in an operation room and in real-time navigation. The Polaris optical tracker (NDI) can be used to reproduce navigation surgical methods. The experimental workflow is as follows: The 2D CT data of the mandibular model in DICOM format are imported to the Slicer program, and rendered into 3D data, as was performed with the Cbyon navigation system. Next, registration was performed using the markers attached to the mandibular model (Fig. 6).

**Fig. 7:**
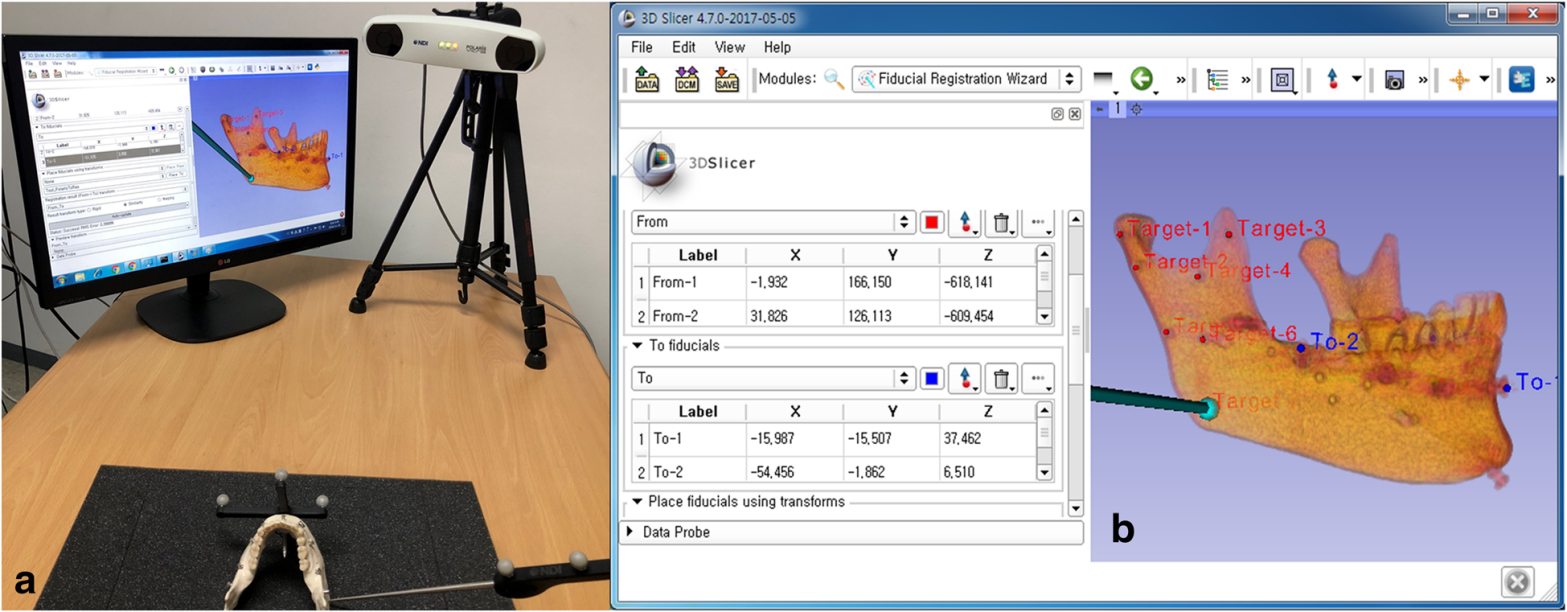
TRE measurement of mandible model on 3D Slicer with Polaris optical tracker. **a** Probing for TRE in the mandibular model. **b** TRE measurement in the 3D Slicer software (e.g., mandibular angle)

After registration, in which the coordinates on the CT images within the 3D Slicer are matched against the actual coordinates on the mandibular model, is complete, the target points are marked on the condylar head and neck, coronoid process, sigmoid notch, posterior border, antilingula, and angle of the mandible on the Slicer to mark the anatomical markers for TRE measurement. The accuracy of navigation was measured according to the number of registration markers ranging from three to seven, as was performed in the Cbyon experiment. The TREs were measured at all target points using a probe. Therefore, the TREs were measured under five different conditions differing in the number of registration reference markers for each of the five models, three times for the left and right sides separately. Measurements were obtained in units of 0.001 mm. The 3D distance errors were calculated based on the X, Y, and Z coordinates, and the TREs were measured for each anatomical site (Fig. 7).

### Statistical analysis

In this study, the mean errors of the TREs measured using two different devices according to the number of markers required during the registration process, which ranged from three to seven, were compared. The analysis of variance was used to test the statistical significance. The SPSS 23.0 software (SPSS Inc., Chicago, IL, USA) was used for the statistical analysis. Values of *p* < 0.05 were considered statistically significant.

## Results

Both experiments were conducted to obtain the TREs at the anatomical locations of the mandible according to the number of registration markers during the navigation process. At both locations, the errors were 2 mm or less. At all anatomical locations, significant differences in the target errors measured by the Cbyon system were found according to the number of registration markers. Significant differences in the target errors measured by the Polaris optical tracker, and 3D Slicer software were found according to the registration markers at the posterior border only. In both groups, the target errors did not decrease as the number of registration markers increased.

### Target registration errors measured by Cbyon navigation surgery equipment according to the number of registration markers

The smallest target errors were measured by Cbyon at five anatomical locations marked by five markers except for the antilingula and the angle of the mandible (Table 1). Significant differences in the number of TREs were found according to the number of registration markers at all anatomical locations. The smallest errors were found at all anatomical locations except for the antilingula and mandibular angle that were registered with five markers. The antilingula site showed the smallest mean error of 1.05 mm when the seven overlay markers were used. However, the error did not decrease according to the number of markers, and a mean error of 1.3 mm was observed when six overlay markers were used. At the mandibular angle, the smallest mean error of 1 mm was found when four overlay markers were used. A mean error smaller than 0.97 mm was observed at the sigmoid notch when five markers were used. Mean errors of 1 mm or greater were observed at all other sites. The greatest mean registration error of 1.7 mm was found at the condyle head when four markers were used.

**Table 1:** Target registration error of mandible in the Cybion navigation surgery device according to the number of registration marker (Mean ± SD)

Anatomic landmarks for measurement	Number of registration marker (mm)					*P*-value
	3	4	5	6	7	
Condyle head (*n* = 100)	1.66 ± 0.62	1.70 ± 0.63	1.45 ± 0.75	1.68 ± 0.69	1.50 ± 0.58	0.015*
Condyle neck (*n* = 100)	1.41 ± 0.60	1.51 ± 0.65	1.14 ± 0.66	1.61 ± 0.71	1.32 ± 0.60	< 0.001*
Coronoid process (*n* = 100)	1.18 ± 0.54	1.24 ± 0.46	1.04 ± 0.39	1.28 ± 0.55	1.23 ± 0.48	0.007*
Sigmoid notch (*n* = 100)	1.15 ± 0.60	1.17 ± 0.47	0.97 ± 0.48	1.30 ± 0.57	1.05 ± 0.60	< 0.001*
Posterior border (*n* = 100)	1.25 ± 0.49	1.32 ± 0.57	1.11 ± 0.53	1.26 ± 0.51	1.17 ± 0.49	0.046*
Antilingula (*n* = 100)	1.13 ± 0.53	1.10 ± 0.47	1.13 ± 0.46	1.30 ± 0.57	1.05 ± 0.43	0.007*
Mandibular anlge (*n* = 100)	1.06 ± 0.52	1.01 ± 0.40	1.12 ± 0.50	1.26 ± 0.51	1.13 ± 0.49	0.009*

*p* < .05*

### Target registration errors measured by 3D slicer software and Polaris optical tracker according to the number of registration markers

In the additional experiment to verify the reliability of the measurements obtained using the Polaris optical tracking system and 3D Slicer S/W, the smallest target errors were found at five anatomical locations excluding the condylar head and neck when four markers were used (Table 2). In the Polaris optical tracker group, the largest mean error was 1.17 mm or less, which was less than the maximum error of 1.7 mm when Cbyon was used. In this experiment, all registration errors were 1 mm or less when four markers were used. Additionally, significant differences in the magnitude of errors were found according to the number of registration markers at all anatomical sites. Meanwhile, significant *p*-values were found at the posterior border when the Polaris optical tracking system and 3D Slicer S/W were used. At the posterior border, the smallest mean error of 0.68 mm was found when four overlay markers were used. However, at all sites but the posterior border, no significant difference in the accuracy of navigation was found according to the number of registration markers. The number of errors did not decrease as the number of overlay markers increased. At the condylar head and neck, mean errors of approximately 0.8 mm were found when the registration was performed with six markers. At the remaining anatomical sites, mean errors of 0.68–0.95 mm were found when the registration was performed with four markers.

**Table 2:** Target registration error of mandible in the device using 3DSlicer software and Polaris optical tracker according to the number of registration marker (Mean ± SD)

Anatomic landmarks for measurement	Number of registration marker (mm)					*P*-value
	3	4	5	6	7	
Condyle head (*n* = 30)	1.00 ± 0.42	0.92 ± 0.43	1.07 ± 0.43	0.80 ± 0.31	1.10 ± 0.53	0.065
Condyle neck (*n* = 30)	1.09 ± 0.47	0.99 ± 0.40	0.95 ± 0.49	0.87 ± 0.41	1.01 ± 0.33	0.361
Coronoid process (*n* = 30)	1.04 ± 0.47	0.95 ± 0.40	0.99 ± 0.36	1.04 ± 0.41	1.17 ± 0.43	0.353
Sigmoid notch (*n* = 30)	0.85 ± 0.43	0.76 ± 0.44	1.01 ± 0.47	0.96 ± 0.33	0.93 ± 0.46	0.213
Posterior border (*n* = 30)	1.02 ± 0.32	0.68 ± 0.26	0.98 ± 0.37	0.80 ± 0.37	0.92 ± 0.36	0.001*
Antilingula (*n* = 30)	1.11 ± 0.45	0.83 ± 0.45	0.97 ± 0.30	0.96 ± 0.43	0.91 ± 0.41	0.134
Mandibular anlge (*n* = 30)	1.00 ± 0.37	0.90 ± 0.49	0.92 ± 0.35	0.91 ± 0.39	1.05 ± 0.38	0.545

*p* < .05*

## Discussion

Significant differences in the target errors measured by the Cbyon system were found according to the number of registration markers used at all anatomical sites. As mentioned previously, significant differences in the target errors measured by the Polaris optical tracker and 3D Slicer S/W were found according to the number of registration markers at the posterior border only. In both groups, the target errors did not decrease as the number of registration markers increased.

It has known that three nonplanar markers are required for the registration procedure in the navigation surgery [8, 12]. In addition, the registration markers should be placed over a large area around the surgical site, and as close to the surgical site as possible to increase the accuracy when five or more markers are used [13, 14]. Considering with these two factors, the number of markers for tooth image overlay was set from three to seven in this study.

However, more time and effort are required during this process as the number of registration markers increases in the clinical settings. Additionally, the number of markers that can be used for overlays may be limited in a real surgery. Furthermore, different decisions regarding the use of invasive markers and marker locations may be made depending on the surgery [15]. In this study, high TREs were observed at five anatomical locations excluding the condylar head and posterior border when six markers were used in the Cbyon group. Furthermore, large errors were found at the condylar head and posterior border when four markers were used. At the antilingula, the smallest error was found when seven markers were used. At the mandibular angle, the smallest error was found when four markers were used. The smallest errors were found at all anatomical sites excluding the antilingula and the mandibular angle when registration was performed with five markers. These results demonstrate that an increase in the number of registration markers is not associated with a decrease in the TRE, and that a specific number of registration markers might reduce the TREs at each anatomical site.

The following factor can be considered for why the large TRE was measured with four markers at the condylar head, while the smallest TRE was measured with four markers at the mandibular angle. During the registration process, the surgical errors can be reduced by setting markers as close as possible to the surgical site. For example, since the marker used in this experiment is at the alveolar bone area under the tooth in the oral cavity, the error can be measured large when the marker is distant from the surgical site, like condyle head. However, in the case of mandibular angle, not only its overall errors are small but the TREs are also small even with few numbers of markers, because it is close to the landmark. The same explanation can be applicable to other cases, including the case of antilingula. Our study focuses on the relation between TREs and the number of markers in the fixed positions of markers. To make clear the relation between TREs and the positions of markers, further study is necessary to compare the error variation according to the marker position in the same number of markers.

In the group where the Polaris optical tracker and 3D Slicer were used, large TREs were observed when different numbers of markers were used. However, considering that the largest mean error values at each anatomical site ranged from 1.01 mm to 1.17 mm, it is difficult to conclude a number of markers have caused significantly large errors. Because large errors were observed when 3, 5, and 7 markers were used, it is possible that errors occurred when the markers placed at the center of the mandible were used as the registration points. In the experimental group, no significant differences in TREs were found according to the number of registration markers at all anatomical sites except for the posterior border. Therefore, three markers may be sufficient to register the image in the mandibular navigation surgery. However, considering that the smallest target error was observed when four markers were used at five anatomical sites excluding the condylar head and neck, the use of four markers may also be considered.

The differences in the experimental results between the Cbyon and Polaris groups may be attributable to the differences in the optical trackers’ sizes, and the precision in the error measurement, algorithm differences between Cbyon S/W, and 3D Slicer S/W used in the image registration, as well as differences in the methods of image development.

Two methods of image registration exist: marker-based registration and marker-free registration. In marker-based registration, reference markers that were clearly visible and identifiable on the CT scans were used [14]. In a marker-free registration, anatomical landmarks or laser surface scanning of the patient’s anatomical structures were used [15–18]. In this study, screws were placed using an invasive technique. The use of noninvasive markers may also be considered and used in navigation surgery. Further research is required to determine the more accurate registration reference method for different surgical sites and methods of surgery.

Marker-free registration uses anatomic landmarks. It involves the use of dental cusps, or all occlusal surfaces of the teeth as registration markers [15]. Further research on navigation surgeries using these methods may enable their application in surgery.

Many errors can arise during the overlaying of digital images using a computer [8, 19]. These errors include measurement errors that occur in the hardware and software of the CT equipment. Imaging errors can arise at certain image modes and software settings such as the matrix size, slice thickness, and voxel size. Registration errors are the most significant errors in image-guided surgery. Registration errors are errors that occur when coupling the image data with the patient’s actual anatomical parts. Registration errors occur when matching the coordinates of the image data against the patient’s anatomical structures and registering them using image-guided navigation surgery. This study investigates the changes in the errors according to the overlaying method by the numbers of paired markers.

A paired fiducial point is the point at which the given image data matches a patient’s anatomical part. Registration errors include fiducial localization errors (FLEs), fiducial registration errors (FREs), and target registration errors (TREs). Fiducial localization errors (FLEs) are errors that arise during the localization of fiducial reference points. FREs are errors between the registered points when overlapping an image with the registration references. TREs are positional errors between the image and real human body following the registration of the registered points.

An optical navigation system has an accuracy of 0.1–04 mm in marker localization [8]. The optical camera of the system must be warmed 15–30 min before surgery [8]. A typical optical navigation system can track a 100 m × 100 m × 100 m area [8]. Errors can arise when the vector angle between the stereo camera and probe, or another surgical tool is 60° or beyond [8]. The vector angle between the optical tracking camera and surgical tools must be less than 50° to reduce errors. In this study, the probe location, angle, and the size and angular distance of the optical tracker may have affected the results.

Compared with an optical navigation system, an electronic navigation system tracks smaller areas, e.g., 50 cm × 50 cm × 50 cm, and may be subject to errors caused by metal instruments [20].

Technical errors arise when tracking a dynamic RF attached to the patient, surgical tools using an activated tracking camera (luminescence sensing diode), or manually by the operator. When using a probe or a surgical tool that is precalibrated in the navigation system and registered in the system program after attaching it to the direct RF (DRF), a calibration process is required. The tool attached to the DRF is tracked using a camera.

Although the mandible is connected to the cranium by the temporomandibular joint, it can be considered as an independent anatomical structure because the temporomandibular joint undergoes continuous movements. The soft tissue composition of the mandible causes severe movements during surgery where the patient must open and close his/her mouth. As computer-assisted mandibular surgery can allow surgeons to locate anatomical structures that are difficult to view and increase the accuracy of surgery, it has become an interesting topic of research in computer-assisted surgery.

When performing image-guided surgery using a navigation system on the mandible, reference points on the mandible itself must be used rather than establishing an overlaying process necessary for navigation surgery using reference points on the cranium or the maxilla. This is because an opened or retracted mandible during mandibular surgery can lead to unilateral displacement. Therefore, methods of mandibular navigation and image-guided surgery using the reference points of the mandible were considered and used in this study. An RF was affixed on the mentum. A stable fixation and prevention of the RF rotation are necessary. Using an optical tracker camera, the RF was affixed at an appropriate angle such that it would be recognized on the monitor. However, the surgeon’s head, in an attempt to view the surgical site, continually obstructed the RF recognition path, rendering real-time monitoring difficult. Methods to overcome this limitation must be devised. Although the use of navigation systems tends to be time consuming initially due to the learning curve, this may gradually improve with time.

## Conclusions

The target errors did not decrease as the number of marker registrations increased in the two groups when the Cbyon navigation surgery equipment or Polaris optical tracker, and 3D Slicer S/W were used. In the Cbyon group, significant differences in the target errors were observed according to the number of registration markers at all anatomical sites. The smallest errors were observed when five markers were used at five anatomical sites, and when four and seven markers were used at two anatomical sites each. In the group where the Polaris optical tracker and 3D Slicer S/W were used, the smallest error was observed at the posterior border when four registration markers were used. It is important to determine the minimum number of image registration markers at which the smallest TRE would be observed for different surgical sites.

Considering the results of the experiment using Cbyon navigation equipment, it is recommended to use five markers in navigation surgery for condyle head, coronoid process, posterior border, condyle neck and sigmoid notch sites. However, the results of the experiment using the Polaris optical tracker and 3D Slicer S/W represented that the TREs of the remaining anatomical structures excluding the posterior border did not show a significant difference from each other. The measurement results in this paper depend on the experimental equipment and target anatomical site. Therefore, further studies are required to clearly determine the appreciate number of image registration markers for navigation-assisted surgery of the mandible.
